# Mechanism of Hypoxia‐Induced HMGB1 Regulating NLRP3 Inflammasome/Caspase‐1 Pathway‐Mediated Pyroptosis in Myocardial Ischemia Reperfusion Injury Through the Nrf2/HO‐1 Pathway

**DOI:** 10.1111/cns.70661

**Published:** 2025-12-11

**Authors:** Fuzhen Zheng, Licheng Yan, Fei Ren, Wenlong Cai, Yongrong Lan, Hong Chen, Qian Chen, Guoxing Weng

**Affiliations:** ^1^ Shengli Clinical Medical College of Fujian Medical University Fuzhou Fujian China; ^2^ Department of Cardiovascular Surgery Fuzhou University Affiliated Provincial Hospital Fuzhou Fujian China

**Keywords:** Caspase‐1, heme oxygenase‐1, high mobility group box 1 protein, myocardial ischemia–reperfusion injury, NLRP3 inflammasome, nuclear factor erythroid 2‐related factor 2, nuclear translocation, Pyroptosis

## Abstract

**Objective:**

Myocardial ischemia–reperfusion injury (MIRI) represents an inevitable risk event for acute myocardial infarction. We explored the mechanism of hypoxia‐induced high‐mobility group box 1 (HMGB1) promoting MIRI by modulating the NLRP3 inflammasome/Caspase‐1 pathway‐mediated pyroptosis via the Nrf2/HO‐1 pathway.

**Methods:**

In vitro cultured mouse cardiomyocytes were exposed to hypoxia/reoxygenation (H/R) to establish an MIRI cell model, then treated with short hairpin‐HMGB1, a NLRP3 agonist (Nigericin), and a Nrf2 inhibitor (ML385). Cell viability and injury were assessed via MTT and LDH assays. HMGB1 (nuclear/cytoplasm), Nrf2 (nuclear/cytoplasm), HO‐1, NLRP3, ASC, cleaved Caspase‐1, and GSDMD‐N protein levels, and IL‐1β and IL‐18 levels in cell supernatants were determined by western blot and ELISA. HMGB1 and Nrf2 distribution were analyzed by immunofluorescence, with their interaction verified by co‐immunoprecipitation. An MIRI mouse model was developed and treated with HMGB1 Box A for in vivo verification.

**Results:**

H/R induction declined the nuclear HMGB1 protein level and cell viability, and intensified the cytoplasmic HMGB1 protein level, cell damage, and pyroptosis‐related protein and inflammatory cytokine levels, which were averted by HMGB1 knockdown. NLRP3 activation partially reversed HMGB1 knockdown's effect on improving cardiomyocyte pyroptosis. Hypoxia‐induced HMGB1 inhibited Nrf2/HO‐1 activation by interacting with Nrf2. Nrf2/HO‐1 suppression partly counteracted HMGB1 knockdown's suppressive effects on NLRP3 inflammasome activation and pyroptosis. HMGB1 suppressed the Nrf2/HO‐1 axis to enhance NLRP3 inflammasome/Caspase‐1 pathway‐mediated pyroptosis, thereby exacerbating MIRI in vivo.

**Conclusion:**

Hypoxia induces HMGB1's nucleus‐to‐cytoplasm translocation, which binds to Nrf2 to repress Nrf2 nuclear translocation to suppress Nrf2/HO‐1 activation to promote NLRP3 inflammasome/Caspase‐1‐mediated pyroptosis, thereby exacerbating MIRI.

## Graphical abstract

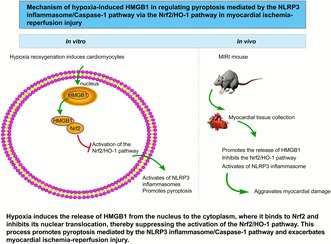

## 1 Introduction

Myocardial ischemia, as a common cardiovascular disorder, has become a significant public health problem due to its high mortality rate and it can lead to irreversible myocardial damage [1]. The prompt restoration of blood flow (reperfusion) in cases of ischemic myocarditis is essential to minimize the infarct size following an acute myocardial infarction [2, 3]. However, reperfusion may result in further myocardial ischemia–reperfusion injury (MIRI), which can lead to structural damage and metabolic dysfunction, including reperfusion arrhythmias, cardiac cell death, myocardial stunning, and microvascular and endothelial dysfunctions [4, 5]. The pathogenesis of MIRI is complex and involves various mechanisms such as apoptosis, reactive oxygen species, inflammation, mitochondrial dysfunction, autophagy, and immune responses [1]. At present, there is no effective treatment available for MIRI [6]. Therefore, it is imperative to explore the mechanisms underlying MIRI to identify novel therapeutic strategies for its treatment.

Pyroptosis is mediated by the formation of macropores in the plasma membrane, which are composed of gasdermin (GSDM)‐family proteins [7]. It is characterized by rapid disruption of the plasma membrane and the release of pro‐inflammatory mediators, including interleukin‐1 (IL)‐1β and IL‐18, and cellular contents [8]. Pyroptosis has been implicated in a range of cardiovascular diseases, such as atherosclerosis, myocardial infarction, and MIRI [9, 10]. Notably, suppression of pyroptosis via genetic or pharmacological interventions has been shown to confer cardioprotective effects under certain conditions [11, 12]. Prior studies have shown that gasdermin D N‐terminal domain (GSDMD‐N) expression is upregulated during the MIRI process [13, 14]. GSDMD is widely recognized as the key executor of pyroptosis [15, 16]. The NOD‐like receptor protein 3 (NLRP3) inflammasome activates the protease Caspase‐1 to evoke GSDMD‐dependent pyroptosis and promote the release of IL‐1β and IL‐18 [17]. Consequently, targeting NLRP3‐mediated pyroptosis may offer innovative approaches for the development of cardioprotective strategies against MIRI.

During myocardial ischemia–reperfusion, various damage‐associated molecular patterns are released, such as reactive oxygen species, adenosine triphosphate, and high‐mobility group box 1 (HMGB1), through the NF‐κB pathway [18]. Among them, as a highly conserved nuclear protein, HMGB1 can be translocated into the cytoplasm and subsequently released into the extracellular space during ischemia and hypoxia, and it plays a pivotal part in the onset of reperfusion injury [19, 20]. The release and secretion of HMGB1 have been reported to diminish cardiomyocyte contractility, trigger hypertrophy and apoptosis in cardiomyocytes, and facilitate activation of the NLRP3 inflammasome [21, 22]. Moreover, inhibition of the HMGB1/TLR4 pathway weakens cardiac myosin activity and curbs the NLRP3 inflammasome/Caspase‐1 axis activation to downregulate intracellular tumor necrosis factor‐alpha, IL‐1β, IL‐18, and reactive oxygen species levels, thereby contributing to the attenuation of the pathological changes of ischemia–reperfusion (I/R) injury [23, 24, 25, 26, 27]. Nevertheless, the exact mechanism of HMGB1 in the pathogenesis of MIRI remains unclear.

As a nuclear transcription factor, nuclear factor erythroid‐2 related factor 2 (Nrf2) regulates the expression of genes involved in antioxidant responses against toxic and oxidative stress to maintain redox homeostasis, and plays a part in various cellular processes, including inflammation and metabolism during MIRI [28] Nrf2 facilitates the transcription of different antioxidant genes, including heme oxygenase‐1 (HO‐1), which is a significant antioxidant enzyme [29]. It has been documented that Sappanone A mitigates MIRI by maintaining redox balance and coordinating cellular antioxidant defenses by modulating Nrf2 [30]. HKL has the capacity to mitigate lipopolysaccharide‐caused acute lung injury by diminishing oxidative stress and curbing NLRP3 inflammasome‐mediated pyroptosis that is partially reliant on Nrf2 activation [31]. Catalpol has been shown to alleviate MIRI by activating the Nrf2/HO‐1 pathway [32]. Moreover, suppression of the Nrf2/HO‐1 pathway results in elevated NLRP3 inflammasome activation in osteoarthritis [33]. Oxycodone mitigates lipopolysaccharide‐caused myocardial injury both in vivo and in vitro by suppressing NLRP3‐mediated pyroptosis through the Nrf2/HO‐1 pathway [34]. The evidence substantiates that the Nrf2/HO‐1 pathway is implicated in NLRP3 inflammasome activation in MIRI. However, further investigation is required to determine whether there are other upstream genes of the Nrf2/HO‐1 pathway on NLRP3 inflammasome/Caspase‐1 pathway‐mediated pyroptosis in MIRI. Based on this context, this study explored the mechanism of hypoxia‐induced HMGB1 facilitating NLRP3 inflammasome/Caspase‐1 pathway‐mediated pyroptosis by curbing Nrf2/HO‐1 pathway activation in MIRI, with the objective to provide new ideas for MIRI treatment.

## 2 Materials and Methods

### 2.1 Ethics Statement

All experimental protocols were reviewed and ratified by the Animal Ethics Committee of Shengli Clinical Medical College of Fujian Medical University, Fuzhou University Affiliated Provincial Hospital and complied with approved protocols strictly. All procedures adhered to internationally recognized guidelines and ethical norms for animal research.

### 2.2 Cell Culture and Treatment

Mouse cardiomyocytes (M6200, Zhongqiao Xinzhou Biotechnology Co. Ltd., Shanghai, China) were exposed to hypoxia/reoxygenation (H/R) to simulate I/R injury in vitro. The normal Dulbecco's modified Eagle medium (DMEM; Gibco, Carlsbad, CA, USA) containing 10% fetal bovine serum was replaced with the glucose‐ and serum‐free DMEM (Gibco). Later, cells were cultivated at 37°C with 5% CO_2_ and 95% N_2_ for 4 h, followed by reoxygenation in a normal medium at 37°C with 95% air and 5% CO_2_ for 8 h.

### 2.3 Cell Grouping and Treatment

Cardiomyocytes were assigned into the following groups: (1) the Control group: without any treatment; (2) the H/R group; (3) the H/*R* + shHMGB1 group: transfected with short hairpin (sh)HMGB1 (Kehao Biotechnology, Xi'an, Shaanxi, China) [35], followed by H/R treatment; (4) the H/*R* + shNC group: transfected with the negative control of shHMGB1 (shNC; Kehao Biotechnology), followed by H/R treatment; (5) the H/*R* + shHMGB1 + *N* group: treated with 20 μmol/L of the NLRP3 activator Nigericin (N; MCE, Monmouth Junction, NJ, USA) [36] while being transfected with shHMGB1 30 min prior to H/R; (6) the H/*R* + shHMGB1 + DMSO group: treated with dimethyl sulfoxide (DMSO; MCE) in the equivalent volume to N and simultaneously transfected with shHMGB1 30 min prior to H/R; (7) the H/*R* + shHMGB1 + ML385 group: treated with 20 μmol/L ML385 (MCE) and simultaneously transfected with shHMGB1 before H/R [37]. Twenty‐four hours after shNC and shHMGB1 transfection, subsequent experiments were carried out.

### 2.4 Experimental Animals

Totally 36 male C57BL/6 mice (2–4 months old; weighing 20 ± 4 g, Vital River Laboratory Animal Technology, Beijing, China) were housed under standard animal facilities (23°C ± 2°C, 60% ± 10% humidity).

### 2.5 Establishment of a Mouse Model of MIRI

As previously described [27, 38], the MIRI mouse model was established. Mice were anesthetized with 2% pentobarbital sodium (PS) (70 mg/kg, P‐010, Sigma‐Aldrich, St. Louis, MO, USA) via intraperitoneal injection and then underwent endotracheal intubation using a mechanically ventilated animal ventilator. The development of the MIRI model was monitored using an electrocardiogram. A thoracotomy was performed at the fourth intercostal space of the left midclavicular line. Next, the left anterior descending (LAD) branches were ligated 1 mm underneath the left atrial appendage for 30 min to induce myocardial ischemia. After untying the ligature, blood flow to the ischemic myocardium was restored for 6 h to induce an MIRI mouse model.

### 2.6 Animal Grouping and Treatment

In the light of the random number table method, mice were allocated into three groups: (1) the Sham group (*n* = 12): mice underwent anesthesia and thoracotomy at the fourth intercostal space of the left midclavicular line. The LAD branches passed underneath 1 mm of the left atrial appendage without ligation; (2) the MIRI group (*n* = 12); (3) the MIRI + HMGB1 Box A group (*n* = 12): each mouse received an intraperitoneal injection of 400 μg of the HMGB1 antagonist Box A [27] prior to IR induction. After anesthesia and thoracotomy, mice were ligated for 45 min and then reperfused for 6 h. Subsequently, mice were sacrificed using 100–150 mg/kg PS via intravenous injection. Heart tissues were harvested, with those from six randomly selected mice in each group for 2,3,5‐triphenyltetrazolium chloride (TTC) staining, and those from the remaining six mice for histological, immunohistochemical, and enzyme‐linked immunosorbent assay (ELISA) analyses.

### 2.7 Assessment of the Left Ventricular Function

M‐mode echocardiography was conducted on anesthetized animals (induced with 2% isoflurane and maintained on 0.8%–1.2% in 100% O_2_). The examination was performed using an HDI‐5000 system with a linear 15 MHz transducer (model CL15‐7; both from ATL‐Phillips, Oceanside, CA, USA) in parasternal short‐axis views at the papillary muscle level [39]. The left ventricular function was assessed by analyzing left ventricular fractional shortening (LVFS), left ventricular end‐diastolic diameter (LVEDd), left ventricular end‐systolic diameter (LVESd), and left ventricular ejection fraction (LVEF) [40, 41].

### 2.8 N‐3‐(4,5‐Dimethylthiazol‐2‐Yl)‐2,5‐Diphenyltetrazolium Bromide (MTT) Assay

Cells cultured in 96‐well plates were fostered with the DMEM containing 5 mg/mL MTT solution (Sigma‐Aldrich) for 4 h at 37°C. After a phosphate‐buffered saline (PBS; 0.1 M) wash, cells in each well were added with 200 μL DMSO solution to dissolve the formazan crystals. Optical density (OD) was measured utilizing a microplate reader (Bio‐Rad, Hercules, CA, USA) at 490 nm to assess cell viability. Finally, the IC_50_ value was calculated using the Pharm statistical software package (Springer Verlag, New York, NY, USA).

### 2.9 Lactate Dehydrogenase (LDH) Assay

The colorimetric LDH Cytotoxicity Assay Kit (A020‐1‐2, Jiancheng Bioengineering Institute, Nanjing, Jiangsu, China) was employed. As per the manufacturer's manuals, sodium hydroxide (NaOH) and pyruvate from the kit were diluted 10‐fold to prepare a 0.4 mol/L NaOH solution and a 0.2 μmol/mL pyruvate standard solution, respectively. Afterwards, cell supernatants or tissue suspensions from each group were obtained as test samples, which were mixed with substrate buffer, coenzyme, 0.2 μmol/mL pyruvate standard solution, and distilled water in the kits in an acceptable manner in the water bath at 37°C for 15 min. Subsequently, samples were mixed with an appropriate amount of 2,4‐dinitrophenylhydrazine in a water bath at 37°C for 15 min, and then mixed with 0.4 mol/L NaOH solution, after which, samples were allowed to stand at ambient temperature for 5 min. OD values at 450 nm were assessed using a microplate reader.

### 2.10 Immunofluorescence (IF)

After fixation and washes with 1× PBS, cells were immersed in 0.01 M citrate buffer (pH 6.0) for heat‐mediated antigen retrieval. Next, the samples underwent fixation with 0.5% Triton X‐100 prepared in 1× PBS at ambient temperature for 10 min, and were cultivated with primary antibodies against Nrf2 (1:200, ab62352, Abcam, Cambridge, UK) and HMGB1 (1:100, ab18256, Abcam) overnight at 4°C. On the next day, samples placed at ambient temperature for 30 min were washed with 1× PBS, and were fostered with DyLight 488‐ or 546‐conjugated goat anti‐rabbit immunoglobulin G (IgG) (Invitrogen, Carlsbad, CA, USA) in the dark at ambient temperature for 1 h. Finally, the samples were mounted in glycerol and stained with 4′,6‐diamidino‐2‐phenylindole (Beyotime, Shanghai, China). Fluorescent images were captured using a BM4000 microscope (Yuyan, Shanghai, China).

### 2.11 Western Blot

Total proteins were isolated from cells using the radio‐immunoprecipitation assay lysis buffer (C1053, Applygen, Beijing, China) and then quantified using a bicinchoninic acid (BCA) kit (Beyotime). Nuclear/cytoplasmic proteins were extracted from cardiomyocytes or myocardial tissues utilizing a kit (78835, Thermo Fisher Scientific, Waltham, MA, USA), with protein quantification implemented via the BCA method. After sodium dodecyl sulfate polyacrylamide gel electrophoresis, samples were transferred onto membranes, which were then blocked with 5% skim milk and fostered with primary antibodies against Nrf2 (1:1000, ab62352, Abcam), HO‐1 (1:2000, ab189491, Abcam), NLRP3 (1:1000, ab263899, Abcam), apoptosis‐associated speck‐like protein containing a CARD (ASC; 1:1000, ab47092, Abcam), cleaved Caspase‐1 (1:1000, #67314, Cell Signaling Technology, Danvers, MA, USA), HMGB1 (1:1000, ab18256, Abcam), GSDMD‐N (1:1000, ab215203, Abcam), β‐actin (1:10000, ab8226, Abcam), and histone H3 (1:1000, ab1791, Abcam) overnight at 4°C. Thereafter, membranes underwent incubation with the secondary antibody goat anti‐mouse IgG (1:1000, ab205719, Abcam) or goat anti‐rabbit IgG (1:1000, ab205718, Abcam) at ambient temperature for 2 h. Fluorescence was visualized using a kit (34580, Thermo Fisher Scientific). A gray‐scale analysis was conducted using ImageJ software (Bio‐Rad).

### 2.12 Co‐Immunoprecipitation (Co‐IP)

Cells lysed using a cell lysis buffer (150 mM NaCl, 5 mM EDTA, 50 mM Tris–HCl, 0.5% [vol/vol] Nonidet P‐40 [NP‐40], 10% [vol/vol] glycerol, pH 7.4; Thermo Fisher Scientific) were supplemented with a mixture of complete protease and phosphatase inhibitors (50×, P1049, Beyotime). Following this, Protein A/G Agarose beads (Fast Flow; Beyotime Institute of Biotechnology) were subjected to incubation with the cell lysis supernatant specific antibody against Nrf2 (#12721, Cell Signaling Technology) or IgG (NC) (1:100, GTX35035, GeneTex, Irvine, CA, USA) at 4°C for 3 h. Next, Agarose beads were washed with 0.1% NP‐40–contained PBS, and bound proteins were eluted. Afterward, Western blot was conducted to assess HMGB1 protein level.

### 2.13 TTC Staining

Mice received intraperitoneal injection of 2% PS (100 mg/kg; P‐010, Sigma‐Aldrich) after the establishment of the I/R model for 7 days. Heart tissues were collected, rinsed with saline, and then sectioned into 4–5 slices (2 mm thickness) underneath the ligation site, after which, sections were fostered in 1% TTC solution (T8877, Sigma‐Aldrich) at 37°C in the dark for 15 min, followed by fixation with 4% paraformaldehyde. Images were captured utilizing a BM4000 microscope (Yuyan) and the myocardial infarct area was analyzed using ImageJ (NIH, Bethesda, MD, USA). The myocardial infarct area was in white, while normal myocardial tissues were stained red.

### 2.14 Hematoxylin–Eosin (HE) Staining

After paraffin embedding, I/R heart tissues were sectioned into 5 mm‐thick slices and stained using an HE staining kit (G1121, Solarbio, Beijing, China). Later, sections were sealed with neutral resin and observed under an inverted microscope (Nikon Ti2, Tokyo, Japan) to evaluate histopathological changes, with images captured.

### 2.15 ELISA

IL‐18 and IL‐1β levels in cell supernatants and heart tissue homogenates were evaluated using ELISA kits (R&D Systems, Minneapolis, MN, USA).

### 2.16 Immunohistochemistry (IHC)

After dewaxing, rehydration, antigen retrieval, and endogenous peroxidase inactivation, sections were fostered with primary antibodies against HMGB1 (1:100, #6893, Cell Signaling), Nrf2 (1:100, #41255, Signalway Antibody, College Park, MD, USA), HO‐1 (1:200, ab189491, Abcam), ASC (1:1000, #bs‐6741R, Bioss, Woburn, MA, USA), GSDMD‐N (1:100, #DF13758, Affinity Biosciences, Liyang, Jiangsu, China), NLRP3 (1:100, #DF7438, Affinity Biosciences), and cleaved Caspase‐1 (1:1000, #AF4022, Affinity Biosciences) overnight at 4°C. After a wash, sections were incubated with the secondary antibody goat anti‐rabbit IgG H&L (HRP) (1:2000, ab205718, Abcam) for 30 min at ambient temperature. Positive sites appeared as brownish‐yellow, and images were analyzed using ImageJ.

### 2.17 Statistical Analysis

Statistical analysis was conducted utilizing GraphPad Prism 9.5.0 (GraphPad Software, San Diego, CA, USA). Normality of data was assessed by the Shapiro–Wilk test. Normally distributed data were reported as mean ± standard deviation (x ± s). Two‐group comparisons were conducted using unpaired Student's *t*‐test, and multi‐group comparisons using one‐way analysis of variance (ANOVA), followed by Tukey's test. A two‐tailed *p* < 0.05 was accepted as indicative of a significant difference.

## 3 Results

### 3.1 H/R Induced HMGB1 Translocation in Cardiomyocytes and Facilitated NLRP3 Inflammasome‐Mediated Pyroptosis

**FIGURE 1:**
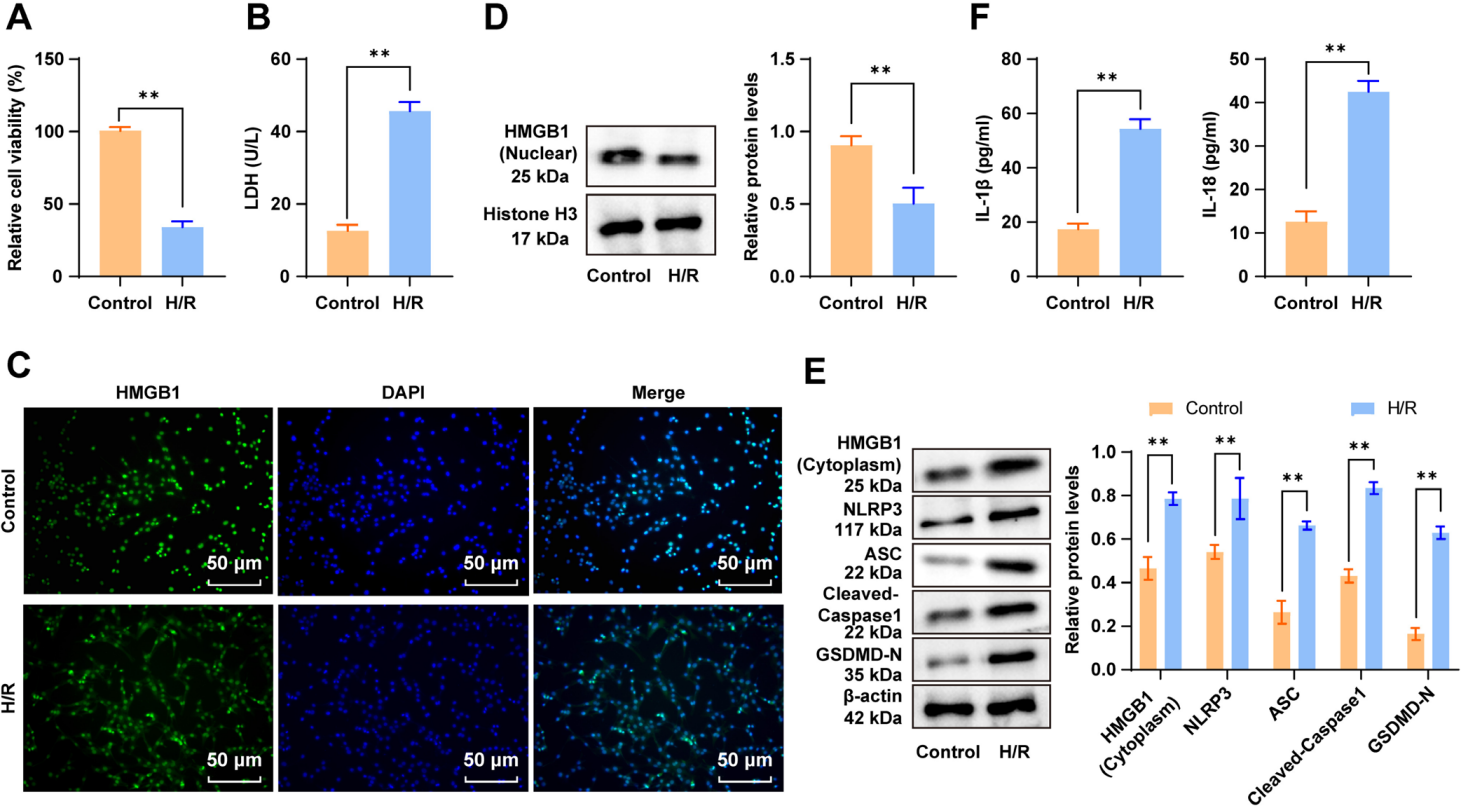
H/R induced the nuclear‐to‐cytoplasmic translocation of HMGB1 and promoted NLRP3 inflammasome‐mediated pyroptosis in cardiomyocytes. (A) Cell viability assessed using the MTT assay; (B) The LDH assay for cellular damage evaluation; (C) The distribution of HMGB1 in cardiomyocytes analyzed by IF; (D) The HMGB1 (nuclear) protein level determined by Western blot; (E) HMGB1 (cytoplasm), NLRP3, ASC, cleaved Caspase‐1, and GSDMD‐N protein levels determined by Western blot; (F) IL‐1β and IL‐18 levels in the cell supernatant measured by ELISA. *n* = 3. Data were expressed as x ± s. Inter‐group comparisons were performed by *t*‐tests. *p < 0.05, **p < 0.01.

To investigate the mechanism of hypoxia‐induced HMGB1 in MIRI, cardiomyocytes were exposed to the H/R treatment to simulate I/R in vitro based on a previous study [42]. As demonstrated by MTT and LDH assay results, compared with the Control group, cardiomyocyte viability was markedly decreased, and cellular damage was remarkably reinforced in the H/R group (Figure 1A,B, all *p* < 0.01). IF and Western blot results revealed a reduced nuclear HMGB1 protein level in the nucleus (Figure 1C,D, all *p* < 0.05) and an elevated cytoplasmic HMGB1 protein level (Figure 1C,E, all *p* < 0.01) after H/R treatment. These results indicated that H/R unveiled HMGB1 translocation from the nucleus to the cytoplasm and exacerbated cardiomyocyte injury. HMGB1 facilitates NLRP3 inflammasome activation‐mediated pyroptosis in H/R‐treated cardiomyocytes [22]. Western blot analysis displayed that protein levels of NLRP3, ASC, cleaved Caspase‐1, and GSDMD‐N were raised in the H/R group versus the Control group (Figure 1E, all *p* < 0.05). With regard to ELISA results, H/R‐treated cell supernatants exhibited upregulated IL‐1β and IL‐18 levels (Figure 1F, all *p* < 0.01). These findings suggested that H/R triggered the nuclear‐to‐cytoplasmic translocation of HMGB1, activated the NLRP3 inflammasome, and promoted cardiomyocyte pyroptosis.

### 3.2 Knockdown of HMGB1 Suppressed H/R‐Induced NLRP3 Inflammasome Activation and Reduced Pyroptosis in Cardiomyocytes

**FIGURE 2:**
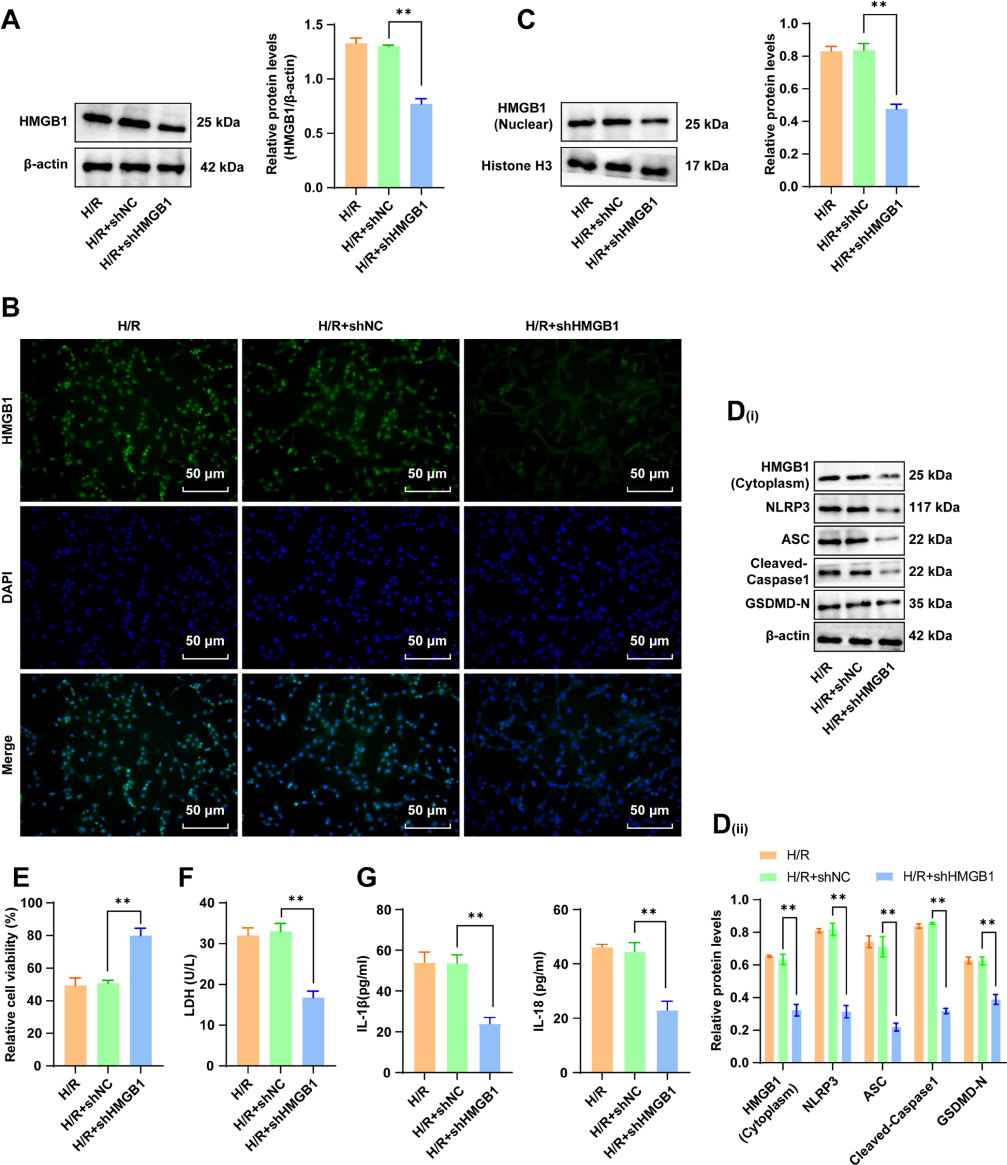
HMGB1 knockdown suppressed H/R‐induced NLRP3 inflammasome activation and reduced pyroptosis in cardiomyocytes. (A) Western blot for measurement of the total HMGB1 protein level; (B) IF for the determination of Nrf2 nuclear translocation; (C) Western blot for determination of the HMGB1 (nuclear) protein level; (D) Western blot for measurement of protein levels of HMGB1 (cytoplasm), NLRP3, ASC, cleaved Caspase‐1, and GSDMD‐N; (E) The MTT assay for cell viability evaluation; (F) The LDH assay for cellular damage assessment; (G) ELISA for the measurement of IL‐1β and IL‐18 levels in the supernatant. *n* = 3. Data were presented as x ± s. Inter‐group comparisons were performed by *t*‐tests. *p < 0.05, **p < 0.01.

Next, H/R‐treated cardiomyocytes were transfected with HMGB1 shRNA to knock down HMGB1 expression (Figure 2A, all *p* < 0.01). Reduced nuclear HMGB1 protein levels (Figure 2B,C, all *p* < 0.01), diminished cytoplasmic HMGB1 protein levels (Figure 2B/D, all *p* < 0.01), enhanced cell viability, reduced cell damage (Figure 2E,F, all *p* < 0.01), downregulated NLRP3, ASC, cleaved Caspase‐1, and GSDMD‐N protein levels (Figure 2D, all *p* < 0.01), and abated IL‐1β and IL‐18 levels in the cell supernatant (Figure 2G, all *p* < 0.01) were observed after HMGB1 knockdown. These findings indicated that the knockdown of HMGB1 suppressed NLRP3 inflammasome activation and reduced pyroptosis to ameliorate H/R‐induced myocardial injury.

### 3.3 NLRP3 Inflammasome Activation Partly Nullified the Ameliorative Effect of HMGB1 Knockdown on H/R‐Induced Cardiomyocyte Pyroptosis

**FIGURE 3:**
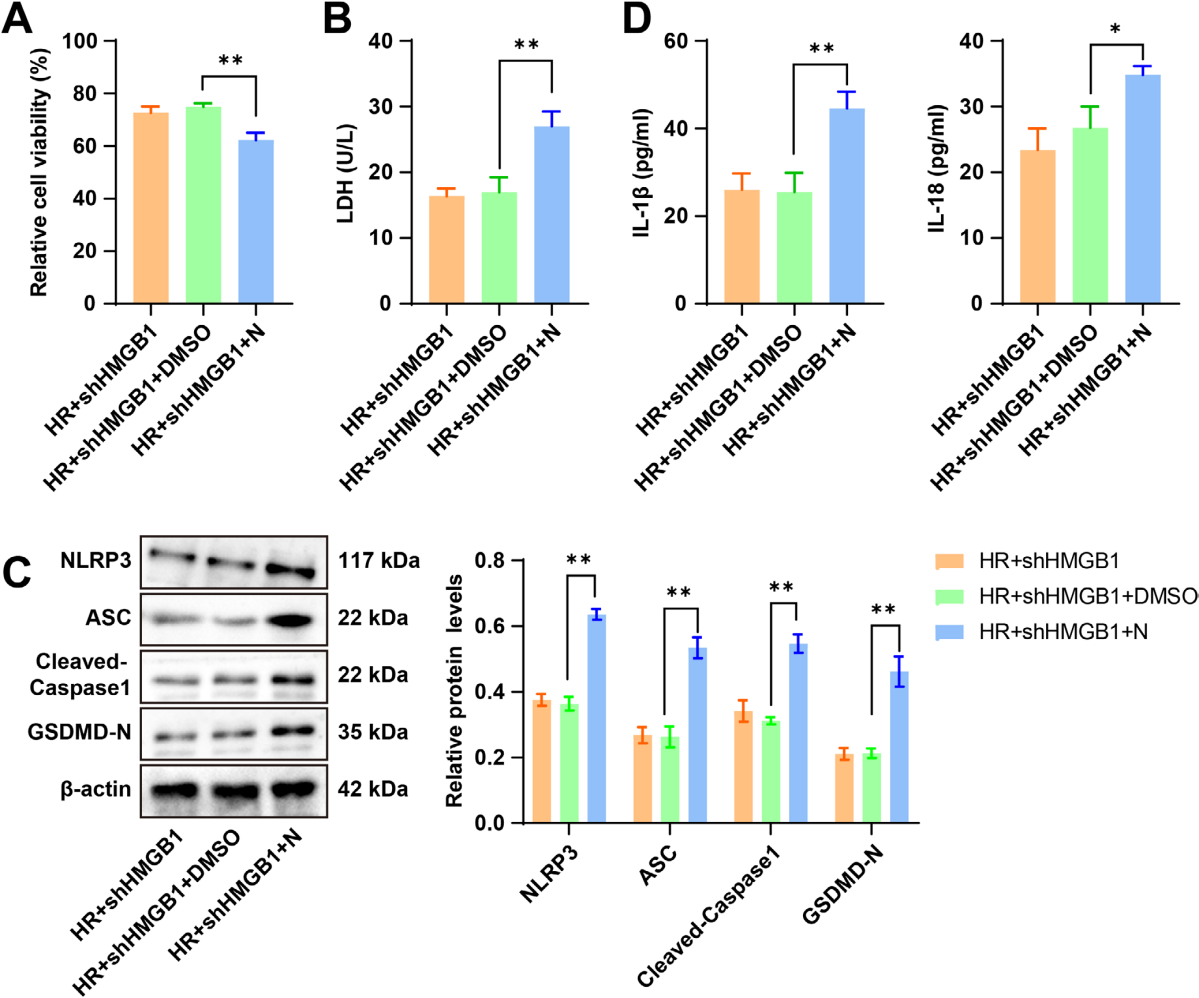
Activation of the NLRP3 inflammasome partially abrogated the ameliorative effect of HMGB1 knockdown on H/R‐induced cardiomyocyte pyroptosis. (A) MTT assay for cell viability assessment; (B) Kit detection of LDH activity to assess cellular damage; (C) Western blot for the measurement of NLRP3, ASC, cleaved Caspase‐1, and GSDMD‐N protein levels; (D) ELISA for the determination of IL‐1β, IL‐18 levels. *n* = 3. Data were reported as x ± s. Multi‐group comparisons were carried out by one‐way ANOVA, and the post hoc analysis by Tukey's multiple comparison tests. *p < 0.05, **p < 0.01.

To confirm the role of HMGB1 in H/R‐induced pyroptosis, we treated cardiomyocytes with 20 μM N 30 min prior to the H/R induction while knocking HMGB1 down. Relative to the H/*R* + shHMGB1 + DMSO group, the H/*R* + shHMGB1 + *N* group exhibited increased NLRP3, ASC, cleaved Caspase‐1, and GSDMD‐N protein levels (Figure 3C, all *p* < 0.01), elevated levels of IL‐1β and IL‐18 in the cell supernatant (Figure 3D, all *p* < 0.01), weakened cell viability, and aggravated cellular damage (Figure 3A,B, all *p* < 0.01). The aforesaid results unraveled that NLRP3 activation nullified the ameliorative effect of HMGB1 silencing on H/R‐induced cardiomyocyte pyroptosis.

### 3.4 Hypoxia‐Induced HMGB1 Subdued the Nrf2/HO‐1 Pathway Activation by Interacting With Nrf2

**FIGURE 4:**
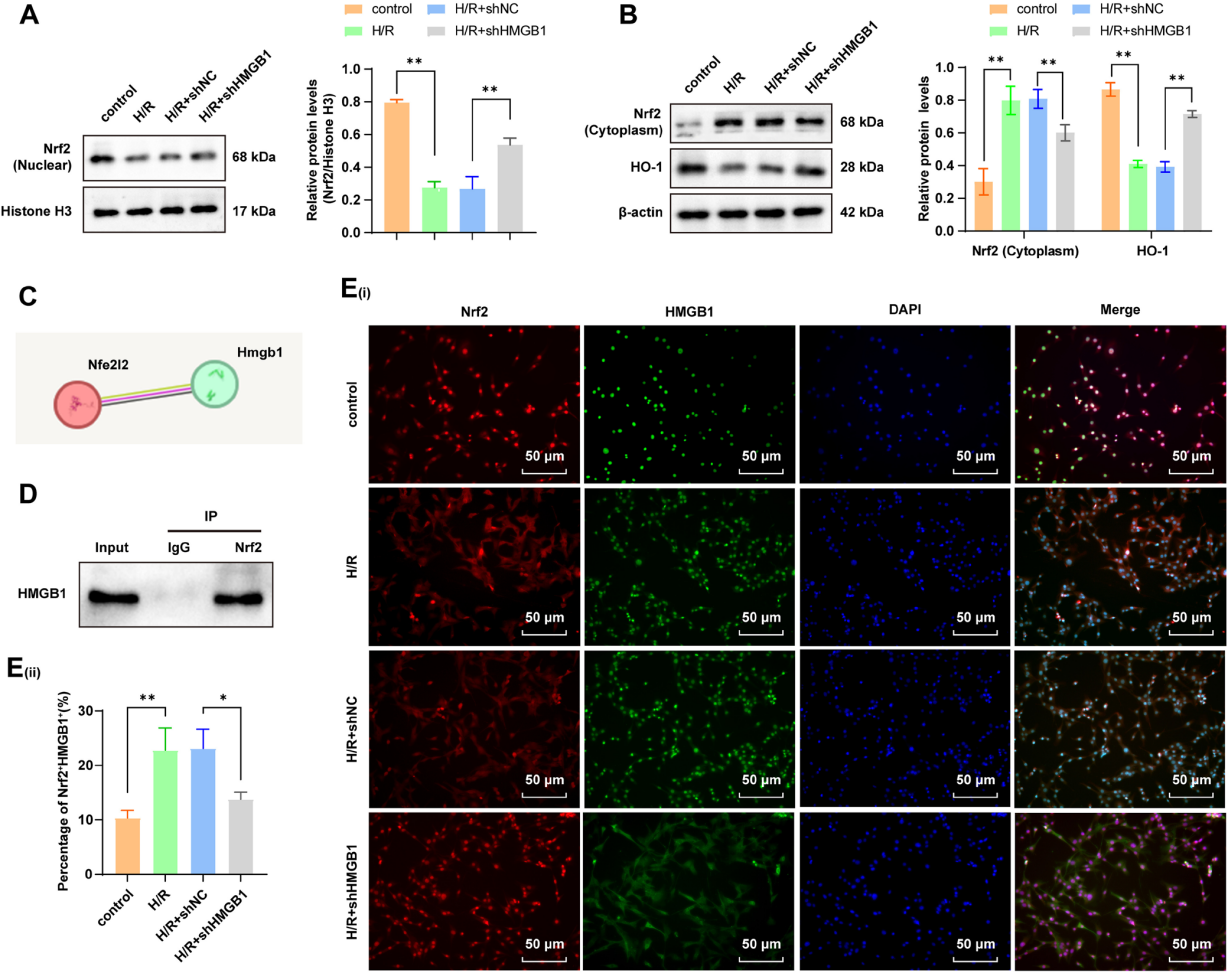
Hypoxia‐induced HMGB1 curbed activation of the Nrf2/HO‐1 pathway by interacting with Nrf2. (A) Western blot to determine the Nrf2 (nuclear) protein level; (B) Western blot to assess Nrf2 (cytoplasm) and HO‐1 expression levels in cells; (C) The STRING database to predict the interaction of HMGB1 with Nrf2; (D) The Co‐IP assay to confirm the interaction of HMGB1 with Nrf2 in cells; (E) IF to determine HMGB1 and Nrf2 expression in cells. *n* = 3. Data were expressed as x ± s. Multi‐group comparisons were carried out by one‐way ANOVA, and post hoc analyses by Tukey's multiple comparison tests. *p < 0.05, **p < 0.01.

As reflected by IF and Western blot analyses, the H/R induction resulted in an increment in cytoplasmic Nrf2 expression, a decrement in the nuclear Nrf2 level, an increase in co‐localization of HMGB1 and Nrf2, and a reduction in the HO‐1 protein level, indicating that H/R suppressed Nrf2 nuclear translocation and inhibited Nrf2/HO‐1 pathway activation; on this basis, further HMGB1 knockdown enhanced Nrf2 nuclear translocation, decreased co‐localization of HMGB1 and Nrf2, reduced the cytoplasmic Nrf2 level, and facilitated Nrf2/HO‐1 pathway activation (Figure 4A,B,E, all *p* < 0.05). The STRING database predicated a potential interaction between HMGB1 and Nrf2 (Figure 4C). We thus speculated that cytoplasmic HMGB1 might interact with the Nrf2 protein to impede nuclear translocation of Nrf2, thereby suppressing Nrf2/HO‐1 pathway activation. The Co‐IP assay confirmed that HMGB1 bound to Nrf2 in the cytoplasm (Figure 4D). IF results demonstrated that the H/R treatment increased the binding of HMGB1 with Nrf2 in the cytoplasm, revealing that H/R prompted the binding of HMGB1 with Nrf2 in the cytoplasm, whereas further HMGB1 knockdown attenuated this binding (Figure 4E). The aforementioned findings suggested that hypoxia‐induced HMGB1 inhibited Nrf2/HO‐1 pathway activation by interacting with Nrf2.

### 3.5 Inhibition of the Nrf2/HO‐1 Pathway Partially Averted the Regulatory Effects of HMGB1 Knockdown on H/R‐Induced NLRP3 Inflammasome Activation and Pyroptosis in Cardiomyocytes

**FIGURE 5:**
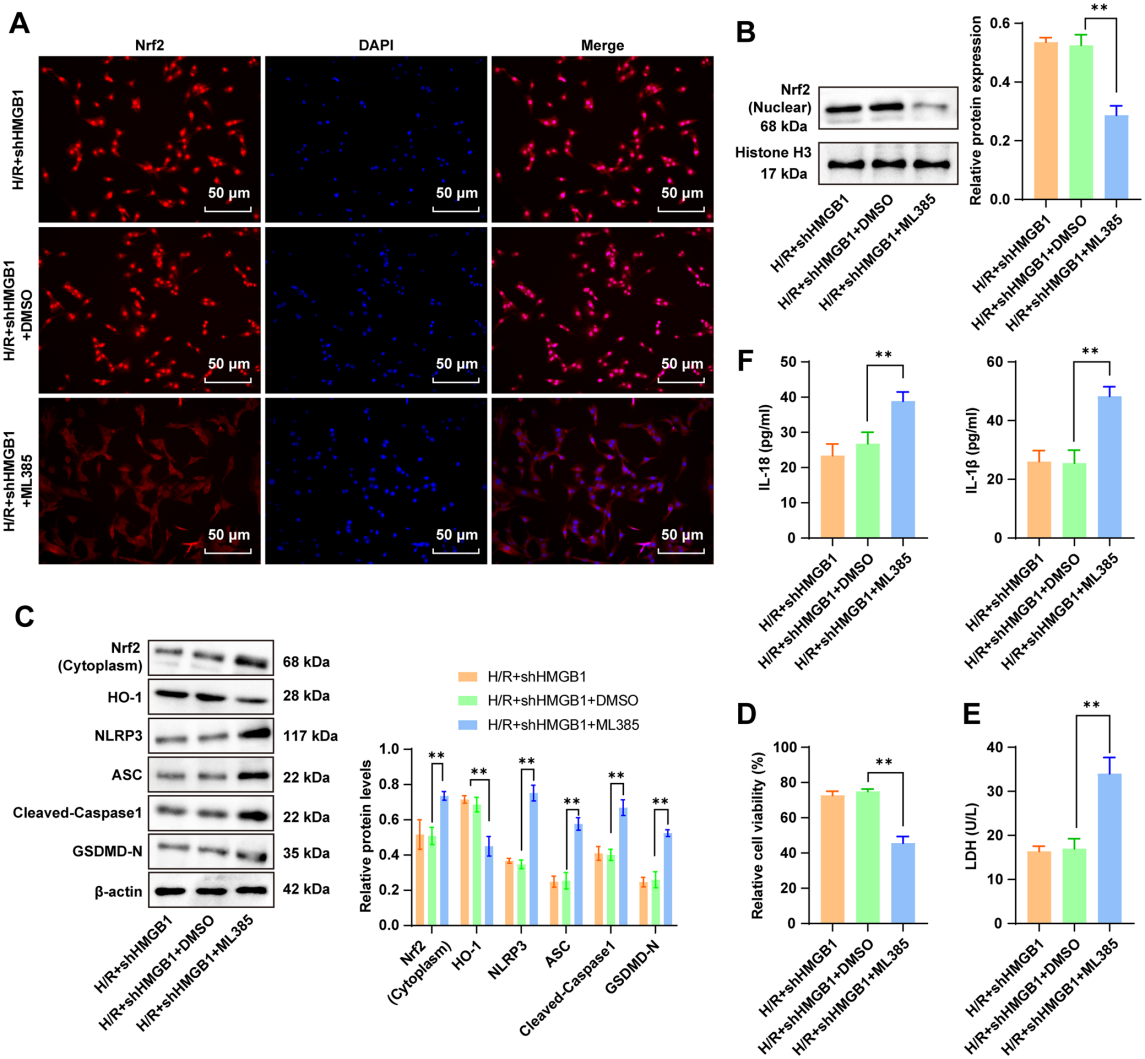
HMGB1 inhibited Nrf2/HO‐1 to regulate the NLRP3 inflammasome/Caspase‐1 pathway‐mediated pyroptosis. (A) Subcellular localization of Nrf2 analyzed by IF; (B) The Nrf2 (nuclear) protein level determined by Western blot; (C) Protein levels of Nrf2 (cytoplasm), HO‐1, NLRP3, ASC, cleaved Caspase‐1, and GSDMD‐N determined by Western blot; (D) Cell viability assessed by the MTT assay; (E) Cellular damage evaluated by the LDH assay; (F) IL‐1β and IL‐18 levels in the supernatant quantified by ELISA. *n* = 3. Data were presented as x ± s. Multi‐group comparisons were carried out by one‐way ANOVA, and post hoc analyses by Tukey's multiple comparison tests. *p < 0.05, **p < 0.01.

Subsequently, H/R‐treated cardiomyocytes were co‐treated with HMGB1 shRNA and the Nrf2 inhibitor ML385. It was found that nuclear translocation of Nrf2 was reduced, cytoplasmic Nrf2 expression was heightened (Figure 5A–C, all *p* < 0.01), the HO‐1 protein level was declined (Figure 5C, all *p* < 0.01), cell viability was diminished, cell damage was aggravated (Figure 5D,E, all *p* < 0.01), while protein levels of NLRP3, ASC, cleaved Caspase‐1, and GSDMD‐N were upregulated (Figure 5C, all *p* < 0.01). Additionally, IL‐1β and IL‐18 levels in the cell supernatant were prominently raised (Figure 5F, all *p* < 0.01). These findings demonstrated that suppression of the Nrf2/HO‐1 pathway partially averted the regulatory effects of HMGB1 knockdown on H/R‐evoked NLRP3 inflammasome activation and pyroptosis in cardiomyocytes.

### 3.6 Hypoxia‐Induced HMGB1 Suppressed the Nrf2/HO‐1 Axis to Enhance the NLRP3 Inflammasome/Caspase‐1 Pathway‐Mediated Pyroptosis, Thereby Exacerbating MIRI in Vivo

**FIGURE 6:**
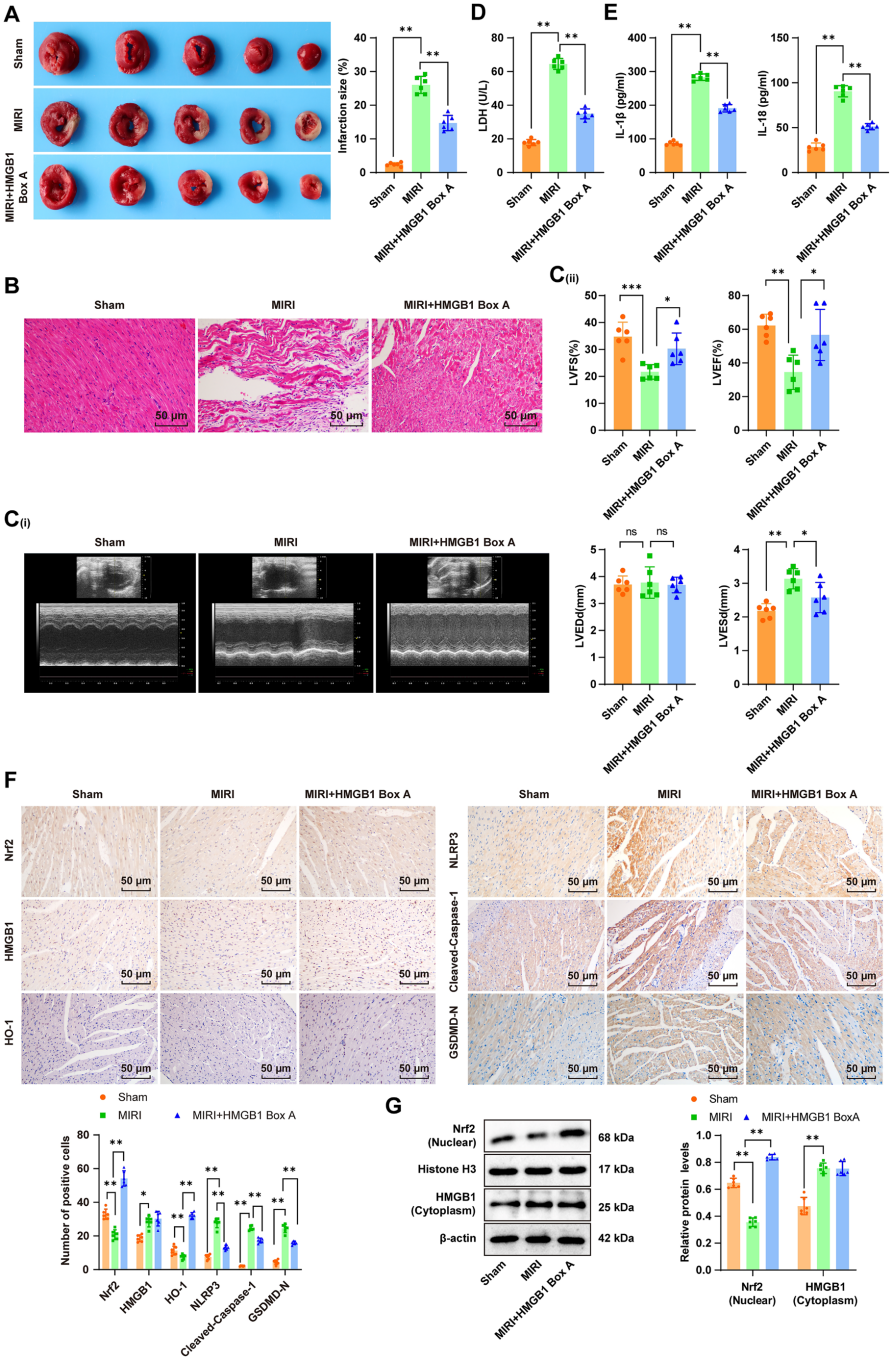
Hypoxia‐induced HMGB1 subdued Nrf2/HO‐1 to regulate the NLRP3 inflammasome/Caspase‐1 pathway‐mediated pyroptosis to promote MIRI. (A) The myocardial infarction area assessed by TTC staining; (B) Histopathological changes in myocardial observed by HE staining; (C) Changes in LVFS, LVEDd, LVESd, and LVEF detected by M‐mode echocardiography; (D) Cellular damage evaluated using the LDH assay; (E) IL‐1β and IL‐18 levels assessed by ELISA; (F) Expression levels of Nrf2, HO‐1, HMGB1, NLRP3, cleaved Caspase‐1, and GSDMD‐N determined by IHC; (G) Nrf2 (nuclear) and HMGB1 (cytoplasm) protein levels measured by Western blot. *n* = 6. Multi‐group comparisons were conducted by one‐way ANOVA, and post hoc analyses by Tukey's multiple comparison tests. *p < 0.05, **p < 0.01.

Finally, as previously described [27], we established an MIRI mouse model. Mice were then administered 400 μg HMGB1 Box A via intraperitoneal injection 1 h before the I/R induction. Relative to the Sham group, the MIRI group exhibited an increased myocardial infarct area (Figure 6A, all *p* < 0.01), a disorganized cardiomyocyte arrangement, enlarged cardiomyocytes, dilated vascular congestion, proliferated inflammatory fibrosis, and aggravated cardiomyocyte damage (Figure 6B,D, all *p* < 0.01). M‐mode echocardiography revealed reductions in LVFS and LVEF and an elevation in LVESd in MIRI mice, indicating impaired left ventricular function (Figure 6C, all *p* < 0.01). Additionally, intensified serum IL‐1β and IL‐18 levels (Figure 6E, all *p* < 0.01), reduced nuclear Nrf2 and HO‐1 expression in myocardial tissues (Figure 6F,G, all *p* < 0.01), and raised cytoplasmic HMGB1, NLRP3, cleaved Caspase‐1, and GSDMD‐N expression (Figure 6F,G, all *p* < 0.01) were observed in the MIRI group versus the Sham group. However, further HMGB1 Box A treatment reversed the above results (Figure 6A–G, all *p* < 0.01). The aforementioned findings suggested that hypoxia‐induced HMGB1 inhibited the Nrf2/HO‐1 pathway to regulate the NLRP3 inflammasome/Caspase‐1 pathway‐mediated pyroptosis, thereby exacerbating MIRI.

## 4 Discussion

MIRI is becoming a prevalent cardiovascular disease with a rising incidence globally and often occurs following the restoration of normal blood flow to the ischemic myocardium after a period of recanalization, which directly contributes to myocardial damage [43]. Notably, HMGB1 is markedly upregulated in the myocardial tissues of MIRI mice, and knockdown of it notably attenuates MIRI [44]. Consequently, this study discovered that hypoxia‐induced HMGB1 inhibited the Nrf2/HO‐1 pathway to regulate the NLRP3 inflammasome/Caspase‐1 pathway‐mediated pyroptosis, thereby exacerbating MIRI.

As a non‐histone DNA‐binding protein, HMGB1 can be released into the extracellular environment by dying cells in response to H/R stimuli [45, 46, 47]. Recent studies have demonstrated that H/R enhances the release of and expression levels of HMGB1 [48, 49]. Furthermore, suppression of I/R‐induced endogenous HMGB1 expression mitigates apoptosis, inflammatory responses, and autophagy, consequently leading to a reduction in the infarct area [50, 51, 52]. It is noteworthy that NLRP3‐mediated pyroptosis has a modulatory role in the onset and development of MIRI [53]. Suppression of NLRP3 inflammasome activation has been demonstrated to limit infarct size and mitigate cardiac dysfunction subsequent to MIRI [54, 55]. Importantly, silencing of HMGB1 results in a reduction of pyroptosis [56]. Silencing of HMGB1 has been shown to reduce pyroptosis caused by hyperhomocysteinemia [57]. The HMGB1 inhibitor effectively diminishes HMGB1 and NLRP3 in macrophages stimulated with palmitic acid, increases cell viability, and decreases pyroptosis [58]. Moreover, repression of the HMGB1/TLR4/NF‐κB/NLRP3 inflammasome pathway protects cardiac myocytes from H/R‐evoked pyroptosis by reducing pyroptosis [27]. In alignment with these findings, we observed that H/R induced HMGB1 translocation, and activated the NLRP3 inflammasome‐mediated pyroptosis of mouse cardiomyocytes, and knockdown of HMGB1 suppressed NLRP3 inflammasome activation and reduced pyroptosis in cardiomyocytes subjected to H/R conditions.

Emerging evidence has suggested that activation of inflammasomes is an indispensable part of MIRI driven by inflammation [59, 60]. Furthermore, activated NLRP3 inflammasomes exacerbate myocardial injury by directly inducing Caspase‐1‐mediated pyroptosis and indirectly facilitating the release of pro‐inflammatory cytokines [61]. In a similar light, our study unveiled that activating the NLRP3 inflammasome partially reversed the ameliorative effect of knockdown of HMGB1 on H/R‐induced cardiomyocyte pyroptosis. Notably, the Nrf2/HO‐1 pathway is crucial for NLRP3 inflammasome activation [62]. Previous evidence has substantiated that HMGB1 knockdown stimulates Nrf2 expression and its downstream targets such as HO‐1, GCLC, NQO‐1, and GCLM [63]. A potential interaction between HMGB1 and Nrf2 was predicted using the STRING database in our study, and the binding of HMGB1 to Nrf2 in the cytoplasm was confirmed through the Co‐IP assay. Our experimental findings revealed that the binding of HMGB1 to Nrf2 in the cytoplasm was enhanced following H/R induction and was reduced after HMGB1 silencing. Innovatively, our study identified that hypoxia‐induced HMGB1 curbed Nrf2/HO‐1 pathway activation by interacting with Nrf2.

The Nrf2/HO‐1 pathway pitches in the process of pyroptosis [64, 65, 66]. It has been documented that deactivation of the Nrf2/HO‐1 pathway leads to increases in the mRNA and protein levels of pyroptosis‐specific markers, including ASC, NLRP3, and Caspase‐1; decreases in the mRNA levels of Nrf2 and HO‐1,; a diminishment in cell viability; and increases in IL‐1β and IL‐18 levels in cardiomyocytes [67]. A study conducted by Chen et al. has unveiled that inhibiting the Nrf2/HO‐1 pathway results in increased activation of the NLRP3 inflammasome, along with upregulation of IL‐1β and IL‐18 expression in osteoarthritis [62]. Interestingly, our study revealed that silencing the Nrf2/HO‐1 pathway partially negated the regulatory effects of HMGB1 knockdown on H/R‐induced NLRP3 inflammasome activation and pyroptosis in cardiomyocytes, evidenced by a reduction in Nrf2 nuclear translocation, an increase in cytoplasmic Nrf2 expression, a decrease in HO‐1 protein levels, a diminishment in cell viability, exacerbation in cell damage, elevations in NLRP3, ASC, cleaved Caspase‐1, and GSDMD‐N protein levels, and increases in IL‐1β and IL‐18 levels in the cellular supernatant after Nrf2 silencing. Caspase‐1 gives an impetus to the maturation of IL‐1β and IL‐18, and is responsible for the activation of the pore‐forming GSDMD [68]. Furthermore, repression of the TXNIP/NLRP3/Caspase‐1 pathway activation provides a protective effect against MIRI [69]. Intriguingly, our study revealed for the first time that hypoxia‐induced HMGB1 suppressed the Nrf2/HO‐1 pathway to regulate the NLRP3 inflammasome/Caspase‐1 pathway‐mediated pyroptosis, thereby aggravating MIRI. Meanwhile, in addition to oxidative stress, numerous molecular events, such as the miR‐450b‐5p/CRYAB axis [70], the FBXW5/ASK1/TRAF6 axis [71], and the miR‐22/FAM49B‐mediated TRAF6/IKK pathway [72], are involved in inflammatory response [70, 71, 72, 73], cell apoptosis [74, 75, 76], and mitochondrial dysfunction [77] pathways, playing critical roles in hepatic/renal/myocardial ischemia–reperfusion injury. This study focused on elucidating the molecular mechanism by which hypoxia induced the translocation of HMGB1 from the nucleus to the cytoplasm, where it bound to Nrf2 and impeded its nuclear translocation to inhibit activation of the Nrf2/HO‐1 antioxidant pathway, thereby promoting NLRP3 inflammasome/Caspase‐1 pathway‐mediated pyroptosis and MIRI. Oxidative stress, as a trigger of pyroptosis, can directly activate the NLRP3 inflammasome through ROS accumulation to induce Caspase‐1‐dependent pyroptosis [78, 79]. The Nrf2/HO‐1 pathway is one of the core pathways regulating oxidative stress and pyroptosis [80]. It has been documented that suppression of NLRP3 inflammasome‐mediated pyroptosis depends on the activation of Nrf2, while suppression of the Nrf2/HO‐1 pathway exacerbates NLRP3 inflammasome activation [31, 34, 62]. This study aims to further elucidate the upstream molecular mechanisms by which the Nrf2/HO‐1 pathway regulates NLRP3 inflammasome/Caspase‐1‐dependent pyroptosis in MIRI, providing new insights for a deeper understanding of this condition.

Taken together, our study highlights that hypoxia induces the nucleus‐to‐cytoplasm translocation of HMGB1, which binds to Nrf2 to repress Nrf2 nuclear translocation to suppress Nrf2/HO‐1 activation to promote NLRP3 inflammasome/Caspase‐1‐mediated pyroptosis, thereby exacerbating MIRI. However, these findings have not yet been verified at the clinical level. Furthermore, the selection of 6 h of IR in this study was primarily based on previous research [81, 82, 83]. The first 6 h following reperfusion is considered a critical time window for the occurrence of cell death (such as apoptosis, ferroptosis, etc.) and inflammatory responses [82]. Furthermore, multiple studies have adopted 6 h of reperfusion as the time point for evaluating drug effects [81, 83]. Additionally, ferroptosis and oxidative stress are substantially activated primarily during the reperfusion phase (rather than the ischemia phase) [84, 85], and 6 h of reperfusion likely encompasses the peak activation of these pathophysiological processes [82]. However, it is undeniable that this single time point fails to capture the dynamic progression of reperfusion injury. Moreover, while this study focused on the role of GSDMD in MIRI, it did not investigate other members of the GSDM family. GSDMD occupies a central and critically important position within the GSDM family, and is widely recognized as the primary executor of pyroptosis [15, 16]. It plays a key role in host defense and immune regulation [86, 87, 88]. GSDMD has also been demonstrated to be closely interrelated with MIRI [13, 14]. However, selectively focusing on GSDMD without systematically comparing the expression patterns and activation mechanisms of different GSDM members in MIRI may lead to the omission of other important regulatory targets. Moreover, the present study only conducted a preliminary investigation into the mechanism by which the inhibition of HMGB1 ameliorated MIRI in mice, and the deeper mechanistic studies regarding the Nrf2/HO‐1 pathway have not been addressed for the time being. In the future, we will further explore the interactions between the NLRP3/Caspase‐1/pyroptosis pathway and other key pathways in MIRI, and develop combined intervention strategies targeting multiple pathways. We will also conduct clinical trials and perform multi‐time point analyses to further validate our findings.

## Author Contributions

All authors contributed to the study conception and design, and all authors commented on previous versions of the manuscript. All authors read and approved the final manuscript. All persons designated as authors qualify for authorship, and all those who qualify for authorship are listed. G.W. is the guarantor of the integrity of the entire study and is responsible for literature research. G.W., F.Z. and L.Y. are responsible for research concepts and study design. G.W., F.Z. and F.R. are responsible for data collection and manuscript preparation. G.W., F.Z. and L.Y. are responsible for manuscript editing. Y.L., L.Y., and W.C. are responsible for the definition of knowledge content and statistical analysis. G.W., F.Z. and L.Y. are responsible for manuscript review. G.W., H.C. and Q.C. are responsible for data analysis. All authors read and approved the final manuscript.

## Disclosure

The authors have nothing to report.

## Ethics Statement

All experimental protocols were reviewed and ratified by the Animal Ethics Committee of Shengli Clinical Medical College of Fujian Medical University, Fuzhou University Affiliated Provincial Hospital and complied with approved protocols strictly. All procedures adhered to internationally recognized guidelines and ethical norms for animal research.

## Consent

The authors have nothing to report.

## Conflicts of Interest

The authors declare no conflicts of interest.

## Data Availability

The data that support the findings of this study are available from the corresponding author upon reasonable request.
